# Macrocarpal C isolated from *Eucalyptus globulus* inhibits dipeptidyl peptidase 4 in an aggregated form

**DOI:** 10.1080/14756366.2017.1396458

**Published:** 2017-11-17

**Authors:** Eisuke Kato, Kazuhiro Kawakami, Jun Kawabata

**Affiliations:** Laboratory of Food Biochemistry, Division of Applied Bioscience, Graduate School of Agriculture, Hokkaido University, Kita-ku, Hokkaido, JapanSapporo

**Keywords:** Dipeptidyl peptidase 4, diabetes, *Eucalyptus globulus*, macrocarpal

## Abstract

Dipeptidyl peptidase 4 (DPP-4) inhibitors are used for the treatment of type-2 diabetes mellitus. Various synthetic inhibitors have been developed to date, and plants containing natural DPP-4 inhibitors have also been identified. Here, 13 plant samples were tested for their DPP-4 inhibitory activity. Macrocarpals A–C were isolated from *Eucalyptus globulus* through activity-guided fractionation and shown to be DPP-4 inhibitors. Of these, macrocarpal C showed the highest inhibitory activity, demonstrating an inhibition curve characterised by a pronounced increase in activity within a narrow concentration range. Evaluation of macrocarpal C solution by turbidity, nuclear magnetic resonance spectroscopy and mass spectrometry indicated its aggregation, which may explain the characteristics of the inhibition curve. These findings will be valuable for further study of potential small molecule DPP-4 inhibitors.

## Introduction

Management of type-2 diabetes mellitus (T2DM) has become an increasingly important task because of the increasing numbers of T2DM patients, as reported by the WHO^1^. Multiple pharmacotherapies have been developed to date^2^^,^^3^, and of these available treatments, GLP-1 receptor agonists and DPP-4 inhibitors have been developed relatively recently which target the incretin system^4^.

DPP-4 is the enzyme responsible for the degradation of GLP-1. Use of a DPP-4 inhibitor extends the half-life of native GLP-1, which is as short as 1–2 min under normal physiological conditions^5^ and therefore permits GLP-1 to restore insulin secretion in T2DM patients, thereby ameliorating their hyperglycaemia. The effect of DPP-4 inhibitors is analogous to that of the use of GLP-1 receptor agonists. However, their chemical nature differs, with GLP-1 receptor agonists being peptide-based, while DPP-4 inhibitors are usually small molecules^4^. Because of this, DPP-4 inhibitors can be administered to patients as orally available drugs.

The oral availability of DPP-4 inhibitors also implies that this class of bioactive compound may be a candidate for discovery in food or plants^6^^,^^7^. We are engaged in evaluating the potential for DPP-4 inhibition by plant-derived substances^8^, and in this communication, 13 plant samples were evaluated for DPP-4 inhibitory activity. *Eucalyptus globulus* was screened for active compounds and macrocarpals were isolated and identified as DPP-4 inhibitors. Among them, macrocarpal C, which is previously reported to have an antibacterial effect^9^ and to inhibit human immunodeficiency virus reverse transcriptase activity^10^, showed a characteristic inhibition curve indicating a distinct inhibitory character from other macrocarpals.

## Methods

### Isolation of macrocarpals A-C

Twenty gram of *E. globulus* leaves were extracted by methanol and partitioned in chloroform/methanol/water in the v/v ratios 4:1:5 to yield 2.85 g extract. This was then separated by silica-gel column chromatography (stepwise elution with hexane/ethyl acetate in the ratios 50:1, 20:1, 10:1, 1:1 and 0:1, and then with methanol alone) to obtain fractions (Fr.) 1–6. Fr. 5 (362 mg), eluted by ethyl acetate, was subsequently separated by silica gel column chromatography (stepwise elution with chloroform/methanol in the ratios 20:1, 10:1, 1:1 and 0:1), preparative TLC (using chloroform/methanol 10:1), and by HPLC [InertSustain C8 (GL Science Co.), gradient elution from 70% methanol/30% water to methanol in 60 min] to obtain 2,4,6-trihydroxy-5-((*R*)-3-methyl-1-((1a*R*,4a*R*,7 *S*,7a*R*,7b*R*)-1,1,7-trimethyl-4-methylenedecahydro-1*H*-cyclopropa[e]azulen-7-yl)butyl)isophthalaldehyde (synonym: macrocarpal C, 1.8 mg). Fr 6 (757 mg), eluted using methanol, was separated by silica-gel column chromatography (first using chloroform/methanol 20:1 containing 1% acetic acid and second using chloroform/methanol 40:1 containing 1% acetic acid), preparative TLC (chloroform/ethyl acetate 3:1 containing 1% acetic acid) and by HPLC [InertSustain C8 (GL Science Co.), gradient elution from 70% methanol/30% water to methanol in 60 min] to obtain 2,4,6-trihydroxy-5-((*R*)-1-((1a*R*,4 *R*,4a*R*,7 *S*,7a*S*,7b*R*)-4-hydroxy-1,1,4,7-tetramethyldecahydro-1*H*-cyclopropa[e]azulen-7-yl)-3-methylbutyl)isophthalaldehyde (synonym: macrocarpal A, 3.1 mg) and 2,4,6-trihydroxy-5-((*S*)-1-((1a*R*,4 *R*,4a*R*,7 *S*,7a*S*,7b*R*)-4-hydroxy-1,1,4,7-tetramethyldecahydro-1*H*-cyclopropa[e]azulen-7-yl)-3-methylbutyl)isophthalaldehyde (synonym: macrocarpal B, 5.4 mg).

### DPP-4 inhibitory activity assay

DPP-4 inhibitory activity was measured using human DPP-4 (Sigma-Aldrich Co.)^11^. The samples, DPP-IV enzyme and the substrate were mixed and incubated for 30 min at 37 °C. The liberated product was quantified using LC-MS to calculate the inhibitory activity (See supporting material for detail). The experiments were carried out in triplicate and representative results are shown in the Figures.

## Results and discussion

### Screening and isolation of DPP-4 inhibitors from *E. Globulus*

Methanol extracts of 13 plant samples were evaluated for their DPP-4 inhibitory activity: *Acer nikoense* branch 15%, *Angelica keiskei* leaf 33%, *Apocynum venetum* leaf 38%, *Citrus limon* pericarp 30%, *Citrus sinensis* pericarp 20%, *E. globulus* leaf 65%, *Glycyrrhiza uralensis* stolon 88%, *Hibiscus sabdariffa* calyx 44%, *Morinda citrifolia* fruit 6%, *Morus alba* leaf 32%, *Myristica fragrans* seed 70%, *Pinus sylvestris* leaf 41% and *Tabebuia impetiginosa* bark 58%. The percentages represent the degree of DPP-4 inhibition measured at the concentration of 0.2 g dry weight/mL for each sample).

**Figure 1.**
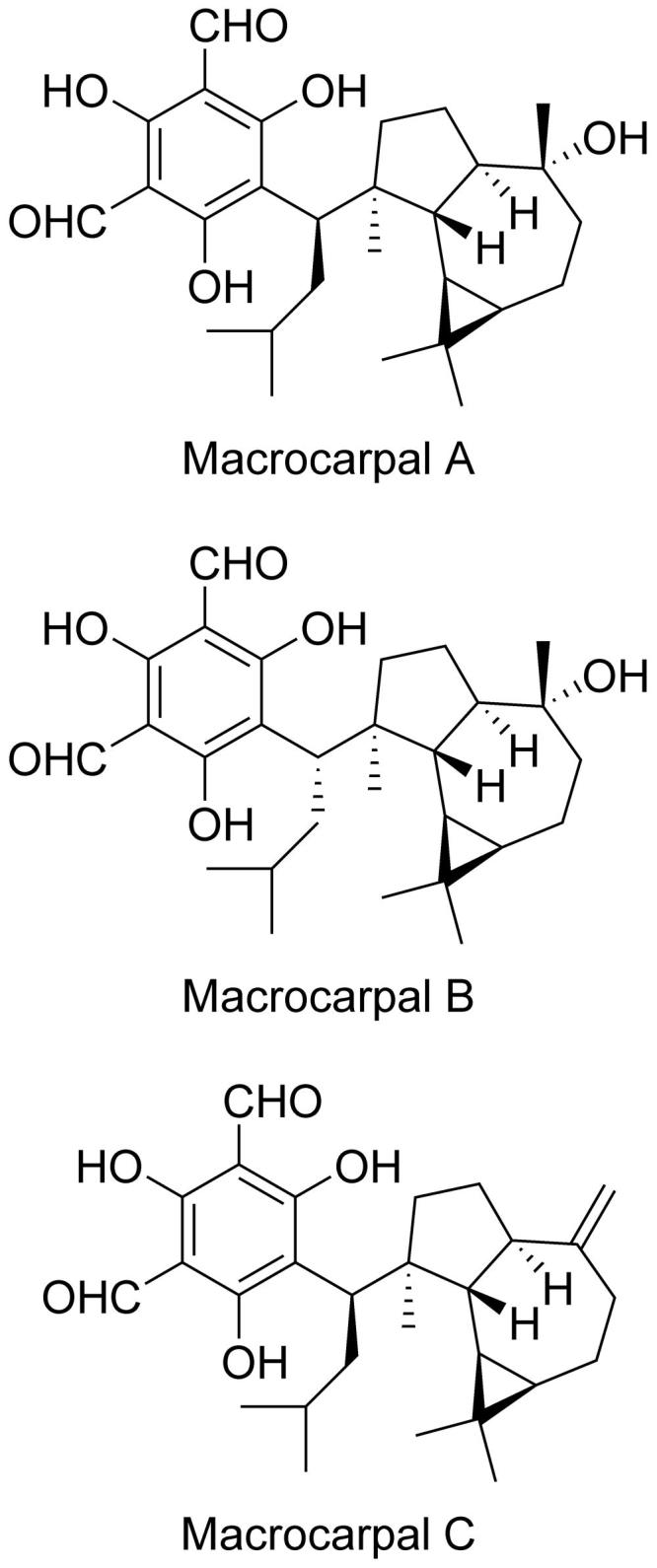
Structure of macrocarpals A–C.

*E. globulus* extract was selected for further study and separated using an activity-guided procedure. Repeated separation by normal phase chromatography and reverse phase HPLC yielded macrocarpals A, B and C (Figure 1), which were identified by NMR and MS analyses, with reference to published data^9^^,^^10^^,^^12^.

### DPP-4 inhibitory activity of macrocarpals

**Figure 2.**
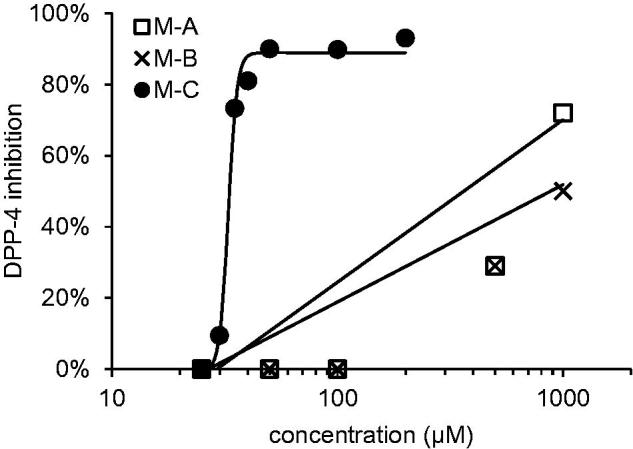
DPP-4 inhibitory activity of macrocarpals A–C M–A: macrocarpal A; M–B: macrocarpal B; M–C: macrocarpal C. Diprotin was used as positive control (30% at 25 µM).

Macrocarpals A–C were tested for their DPP-4 inhibitory activity (Figure 2). Although macrocarpals A and B showed modest activity, with 30% inhibition at 500 µM for both compounds, macrocarpal C showed potent activity, with 90% inhibition at 50 µM. The inhibition curve for macrocarpal C differed markedly from those of macrocarpals A and B. For macrocarpals A and B, linear increases in DPP-4 inhibition were observed, while macrocarpal C showed a marked increase in inhibitory activity within a narrow concentration region, exhibiting almost no activity below 30 µM, but potent activity above 35 µM.

The structures of macrocarpals A–C are quite similar; therefore, the binding of the individual molecules to DPP-4 and the associated inhibition curves would not be expected to differ significantly. The threshold for inhibitory activity by macrocarpal C therefore suggests that more than one molecule may be involved in the inhibition, and aggregation was suspected in the first instance.

### Assessment for macrocarpal C aggregation

To evaluate the tendency for macrocarpal C to form aggregates in solution, the turbidity of solutions of macrocarpals A–C in 10% DMSO, the solvent used for the DPP-4 inhibitory activity assays, were compared. As shown in Table 1, macrocarpal C was the only compound to cause turbidity. However, the conditions for the DPP-4 inhibitory activity assay included pH control by the buffer (pH 8) and under the same conditions, the solution of macrocarpal C remained clear, presumably due to higher solubility of the phenol groups at pH 8 (data not shown). Therefore, NMR and ESI-MS analysis were also undertaken to evaluate the aggregation of macrocarpals A–C.

**Table 1.** Turbidity of macrocarpals A–C dispersed in 10% DMSO.

	Concentration (µM)					
Compound	0	10	50	100	500	1000
macrocarpal A	−1	−1	0	5	2	13
macrocarpal B	0	0	1	1	3	12
macrocarpal C	1	0	16	41	349	763

Data are A_600nm_ ×10^3^.

**Figure 3.**
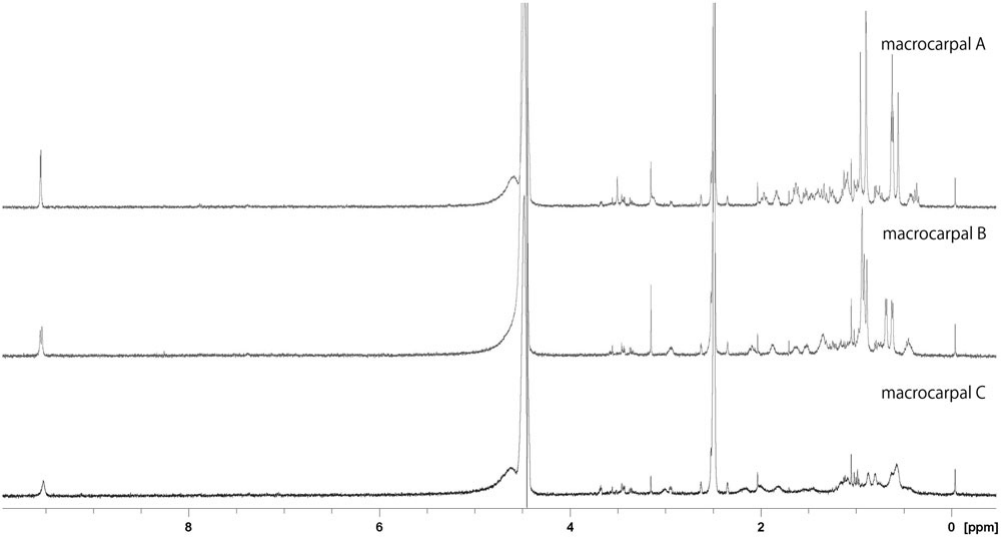
^1^H-NMR spectrum (500 MHz, 37 °C) of macrocarpals A–C in buffered DMSO solution.

Figure 3 shows the ^1^H-NMR spectra for macrocarpals A–C (100 µM) dissolved in 10% DMSO/20 mM phosphate buffer (pH 8) containing 100 mM sodium chloride. All three compounds possess methyl groups, and on the macrocarpal A and B spectra, these signals are clearly shown within 0.5–1.5 ppm. In contrast, the same signals in macrocarpal C are broader and are not clearly distinguishable from others. In addition, ESI-MS analysis of macrocarpal C yielded a peak at *m*/*z* = 907.54 [2 M-H], which was not observed for macrocarpal A and B.

Line broadening on NMR is a well-known sign of limited bond rotation^13^, and ESI-MS represents a soft ionisation mode that gives information about interacting products^14^. The broadened NMR signals and the [2 M-H] peak observed using ESI-MS analysis therefore suggest the interaction of two or more macrocarpal C molecules under the conditions used for the enzyme reaction, which may help to explain the nature of the inhibition curve obtained for macrocarpal C.

## Conclusions

Macrocarpals A–C were identified as DPP-4 inhibitors within *E. globulus*. Macrocarpal C showed the highest activity and demonstrated a characteristic inhibition curve, which is likely the result of self-aggregation of the compound. Inhibition of DPP-4 by this compound is a novel finding, as is the nature of the inhibition curve. This knowledge should be valuable for further study of potential small molecule DPP-4 inhibitors.

## Supplementary Material

IENZ_1396458_Supplementary_Material.pdf
