# Supplementary material for: Macrocarpal C isolated from Eucalyptus globulus inhibits dipeptidyl peptidase 4 in an aggregated form
Source: J Enzyme Inhib Med Chem. 2017 Nov 17;33(1):106–9. doi: 10.1080/14756366.2017.1396458 (PMC6009884; doi:10.1080/14756366.2017.1396458)
Supplement: IENZ_1396458_Supplementary_Material.pdf [file IENZ_A_1396458_SM7801.pdf]

Supplementary material

Macrocarpal C isolated from *Eucalyptus globulus* inhibits dipeptidyl peptidase 4 in an aggregated form

Eisuke Kato*, Kazuhiro Kawakami, Jun Kawabata

Laboratory of Food Biochemistry, Division of Applied Bioscience, Graduate School of Agriculture, Hokkaido University, Kita-ku, Sapporo, Hokkaido 060-8589, Japan

*Corresponding author: Dr. Eisuke Kato

Laboratory of Food Biochemistry, Division of Applied Bioscience, Graduate School of Agriculture, Hokkaido University, Kita-ku, Sapporo, Hokkaido 060-8589, Japan.

Tel/Fax: +81 11 706 2496; E-mail: eikato@chem.agr.hokudai.ac.jp

Contents

General experimental information 2
Preparation of the plant extracts 2
DPP-4 inhibitory activity assay 2
Measurement of turbidity 3
Compound data 4
NMR spectrum 5
ESI-MS spectrum 8

General experimental information

Commercially available chemicals were purchased from Wako Pure Chemical Industries, Ltd. unless otherwise stated. A Waters LCT Premier spectrometer (Waters Co.) combined with a Waters Acquity UPLC system (Waters Co.) was used for a liquid chromatography-mass spectrometry (LC-MS) analysis. A Bruker AMX 500 (Bruker BioSpin K.K.) was used to obtain nuclear magnetic resonance (NMR) spectra, using residual solvents as internal standards (methanol-d_4: ^{1}H 3.31 ppm, ^{13}C 49.0 ppm; dimethyl sulfoxide (DMSO)-d_6: ^{1}H 2.49 ppm). A Jeol JMS-T100GCV (Jeol Ltd.) was used for field desorption-mass spectrometry (FD-MS) analysis. A Synergy™ MX (Bio-tech Instruments Inc.,) microplate reader was used to measure absorbance.

Preparation of the plant extracts

Dried plant samples were purchased from a commercial supplier (Hyakka-Saen.Co.Ltd, Fukuoka, Japan). Plant samples were powdered and extracted twice using methanol for 24 h each. The extract was evaporated, and the residue was partitioned in chloroform/methanol/water in the v/v ratios 4:1:5. The obtained chloroform layer was washed using 1% sodium chloride solution, evaporated and dissolved in DMSO.

DPP-4 inhibitory activity assay

DPP-4 inhibitory activity was measured using human DPP-4 (Sigma-Aldrich Co.). The samples, dissolved in 10 µl DMSO, tris buffer (30 µL; 20 mM Tris, 100 mM sodium chloride, 1 mM EDTA, pH 8.0) and DPP-IV enzyme solution (10 µL) were mixed and pre-incubated at 37°C. The substrate solution (50 µL; 200 µM glycyl-L-proline 4-methylcoumaryl-7-amide dissolved in tris buffer,

Peptide Institute, Inc.) was added and incubated for 30 min at 37°C. The reaction was stopped by adding 10 µL of sitagliptin solution (50 µM, Cayman Chemical) dissolved in DMSO, and 7-amino-4-trifluoromethylcoumarin (AFC, Sigma-Aldrich Co.) was used as the internal standard. The reaction mixture was diluted five-fold using methanol and the liberated 7-amino-4-methylcoumarin (AMC) was quantified using LC-MS [column: InertSustain C18 (GL Science Co., 2.1×100 mm); mobile phase: 65% methanol/35% water containing 0.1% formic acid; flow rate: 0.2 mL/min; detection: positive mode, m/z = 176.07 for AMC, m/z = 230.04 for AFC]. The negative control contained DMSO in place of sample solution, and diprotin A (25 µM; Peptide Institute, Inc.) was used as a positive control. The experiments were carried out in triplicate and representative results are shown in the Figures.

Measurement of turbidity

Solutions of each sample in DMSO were diluted 10-fold in water and incubated for 30 min at 37°C. The absorbance of the resulting mixture was measured at 600 nm.

Compound data

macrocarpal A: ^{1}H-NMR (500 MHz, methanol-d_4) 0.53-0.59 (2H, m), 0.75 (3H, s), 0.81 (6H, d, J = 6.6 Hz), 0.90-1.02 (2H, m), 1.08 (3H, s), 1.10 (3H, s), 1.12 (3H, s), 1.28-1.35 (2H, m), 1.45-1.58 (2H, m), 1.64-1.73 (3H, m), 1.76-1.87 (2H, m), 1.98-2.04 (1H, m), 2.26 (1H, td, J = 4.0, 12.8 Hz), 3.36 (1H, dd, J = 4.1, 12.6 Hz), 9.84 (1H, s), 9.86 (1H, s) ppm; ^{13}C-NMR (125 MHz, methanol-d_4) 18.2, 20.4, 20.8, 21.6, 22.0, 23.0, 25.2, 25.8, 27.3, 28.6, 29.2, 29.6, 36.1, 36.5, 36.7, 45.2, 45.5, 49.8, 55.9, 76.8, 103.8, 109.6, 109.7, 174.1, 174.8, 182.4, 191.8, 192.1 ppm; FD-MS (positive) m/z: $[M]^+$ Calcd for $C_{28}H_{40}O_6$ 472.2825, found 472.2839; $[\alpha]^{24}_{D}$ = -72.0 (c=0.26, ethanol).

Macrocarpal B: ^{1}H-NMR (500 MHz, methanol-d_4) 0.54-0.60 (1H, m), 0.64 (1H, t, J = 9.8 Hz), 0.81 (3H, d, J = 5.6 Hz), 0.86 (3H, d, J = 6.0 Hz), 0.95-1.05 (2H, m), 1.08 (3H, s), 1.11 (3H, s), 1.14 (3H, s), 1.12 (3H, s), 1.17-1.36 (3H, m), 1.44-1.72 (5H, m), 1.76-1.83 (1H, m), 2.00-2.07 (1H, m), 2.36 (1H, td, J = 3.5, 12.6 Hz), 3.19 (1H, dd, J = 2.5, 13.2 Hz), 9.83 (1H, s), 9.85 (1H, s) ppm; ^{13}C-NMR (125 MHz, methanol-d_4) 17.7, 17.8, 20.4, 20.4, 21.6, 22.0, 25.2, 25.5, 27.3, 28.3, 29.5, 31.2, 37.1, 40.8, 41.6, 45.3, 49.9, 50.5, 58.9, 76.6, 104.3, 109.5, 109.7, 174.3, 174.8, 182.4, 191.8, 192.1 ppm; FD-MS (positive) m/z: $[M]^+$ Calcd for $C_{28}H_{40}O_6$ 472.2825, found 472.2842; $[\alpha]^{24}_{D}$ = -23.5 (c=0.45, ethanol).

macrocarpal C: ^{1}H-NMR (500 MHz, methanol-d_4) 0.63-0.69 (2H, m), 0.77 (3H, d, J = 6.0 Hz), 0.78 (3H, d, J = 6.9 Hz), 0.80 (3H, s), 0.92-0.96 (1H, m), 1.00 (3H, s), 1.08 (3H, s), 1.14-1.22 (2H, m), 1.30-1.40 (2H, m), 1.60-1.68 (1H, m), 1.72-1.80 (1H, m), 1.94-2.04 (2H, m), 2.23-2.39 (4H, m), 3.38 (1H, dd, J = 3.5 Hz, 12.3 Hz), 4.62 (1H, s), 4.68 (1H, s), 10.09 (2H, s) ppm; ^{13}C-NMR (125 MHz, methanol-d_4) 17.7, 21.1, 22.7, 24.1, 25.1, 27.0, 28.3, 28.6, 28.8, 29.6, 29.6, 36.1, 37.5, 38.8, 40.7, 50.0, 51.7, 52.6, 106.4, 106.4, 106.6, 111.1, 156.5, 169.5, 170.7, 171.3, 191.8, 192.1 ppm; FD-MS (positive) m/z: $[M]^+$ Calcd for $C_{28}H_{38}O_5$ 454.2719, found 454.2737; $[\alpha]^{24}_{D}$ = -22.1 (c=0.80, ethanol).

NMR spectrum

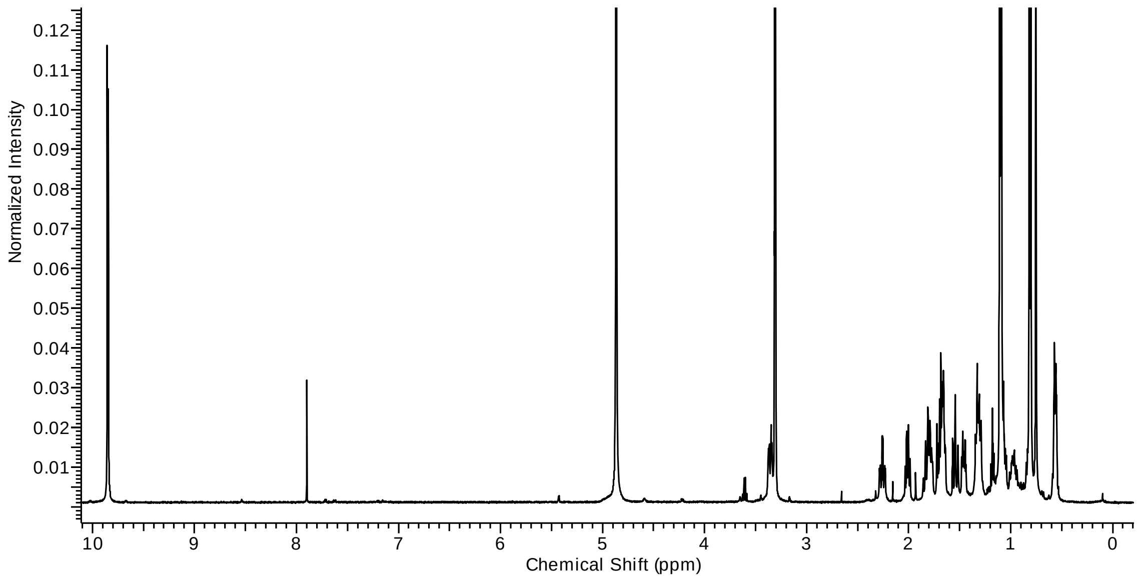

S Fig. 1. ^{1}H-NMR spectrum of macrocarpal A (500 MHz、CD_3OD)

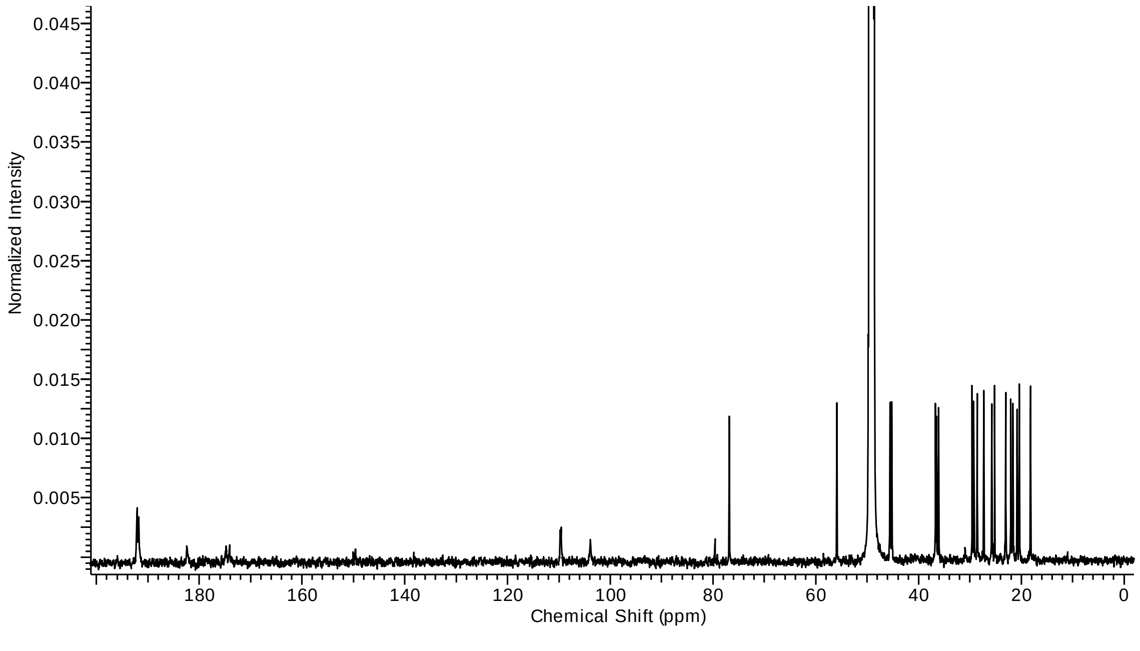

S Fig. 2. ^{13}C-NMR spectrum of macrocarpal A (125 MHz、CD_3OD)

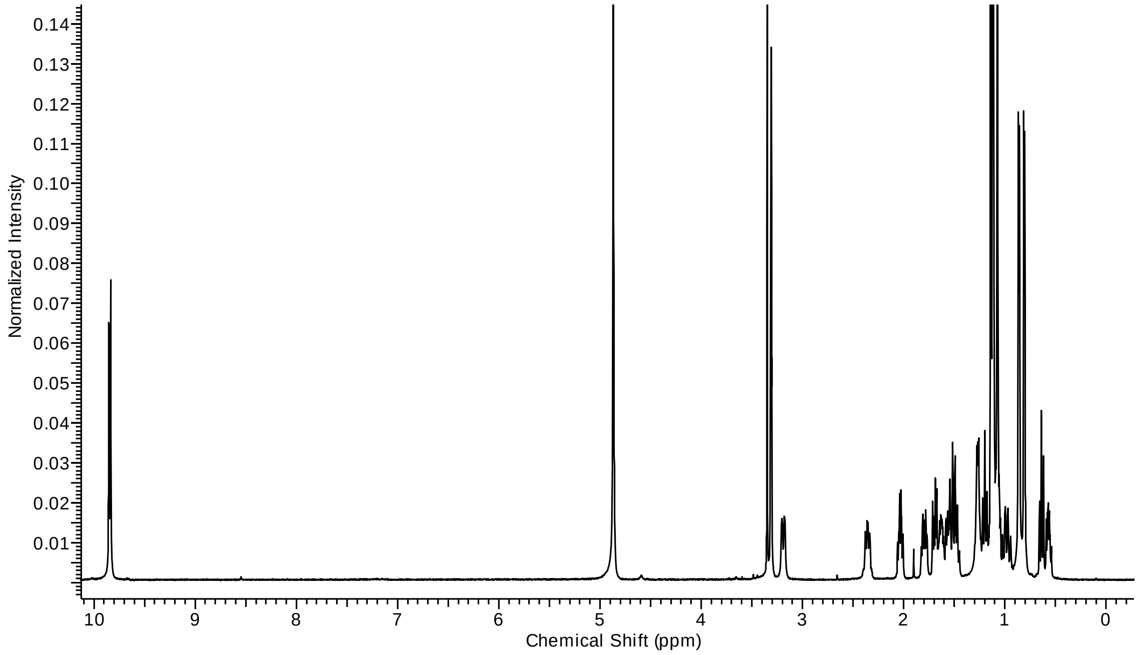

S Fig. 3. ^{1}H-NMR spectrum of macrocarpal B (500 MHz、CD_3OD)

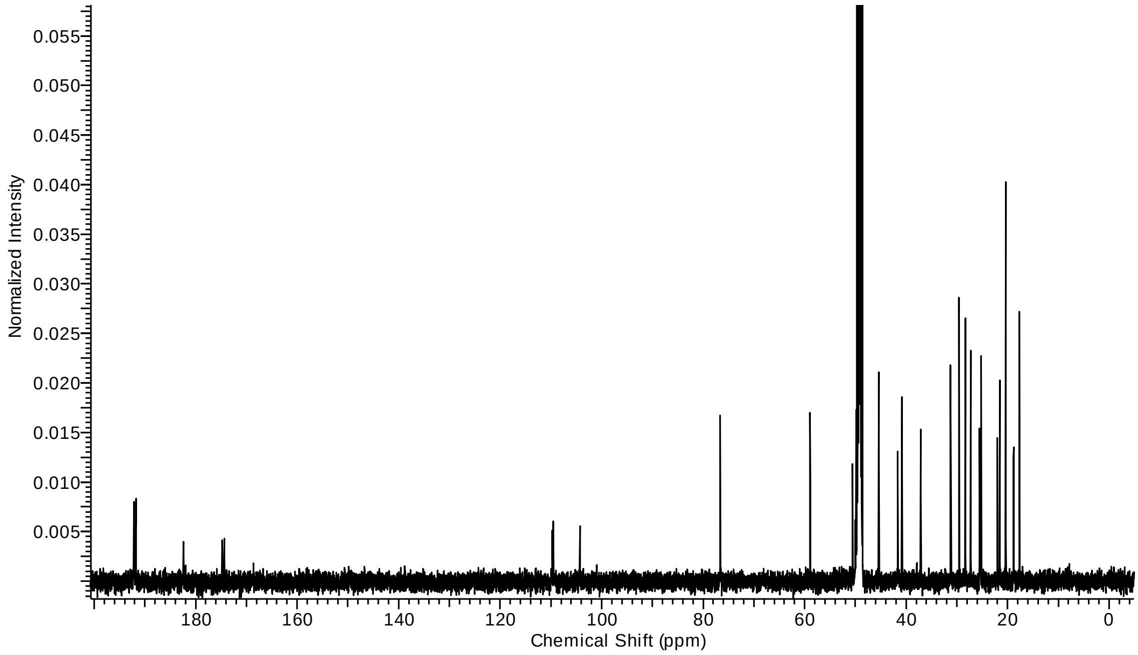

S Fig. 4. ^{13}C-NMR spectrum of macrocarpal B (125 MHz、CD_3OD)

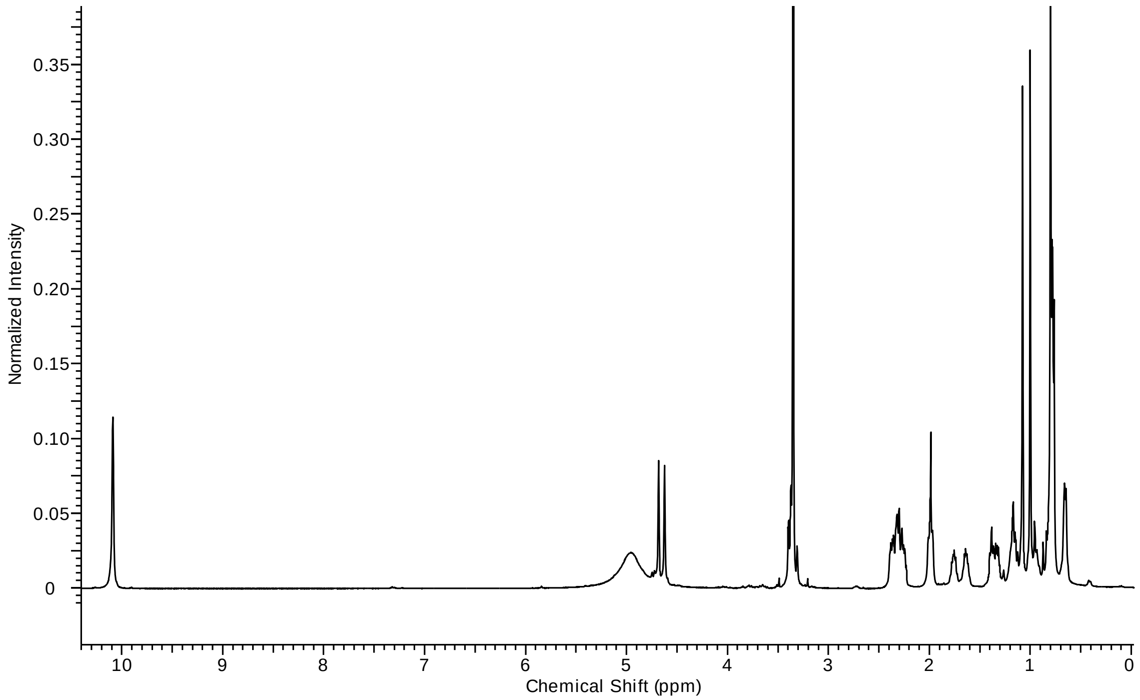

S Fig. 5. ^{1}H-NMR spectrum of macrocarpal C (500 MHz、CD_3OD)

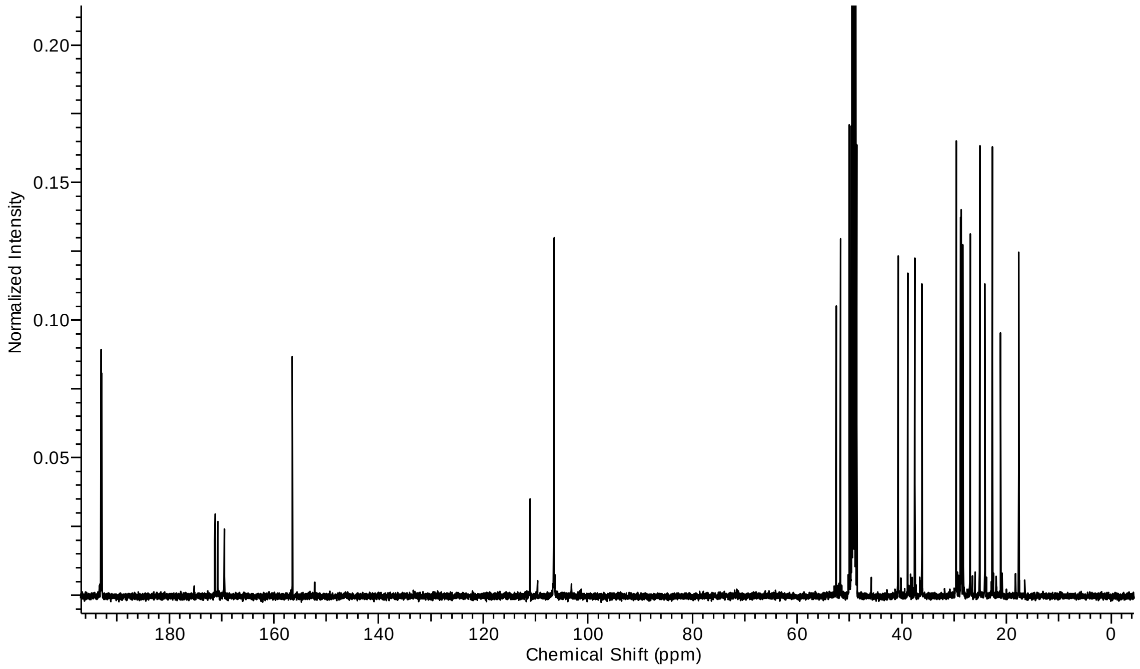

S Fig. 6. ^{13}C-NMR spectrum of macrocarpal C (125 MHz、CD_3OD)

ESI-MS spectrum

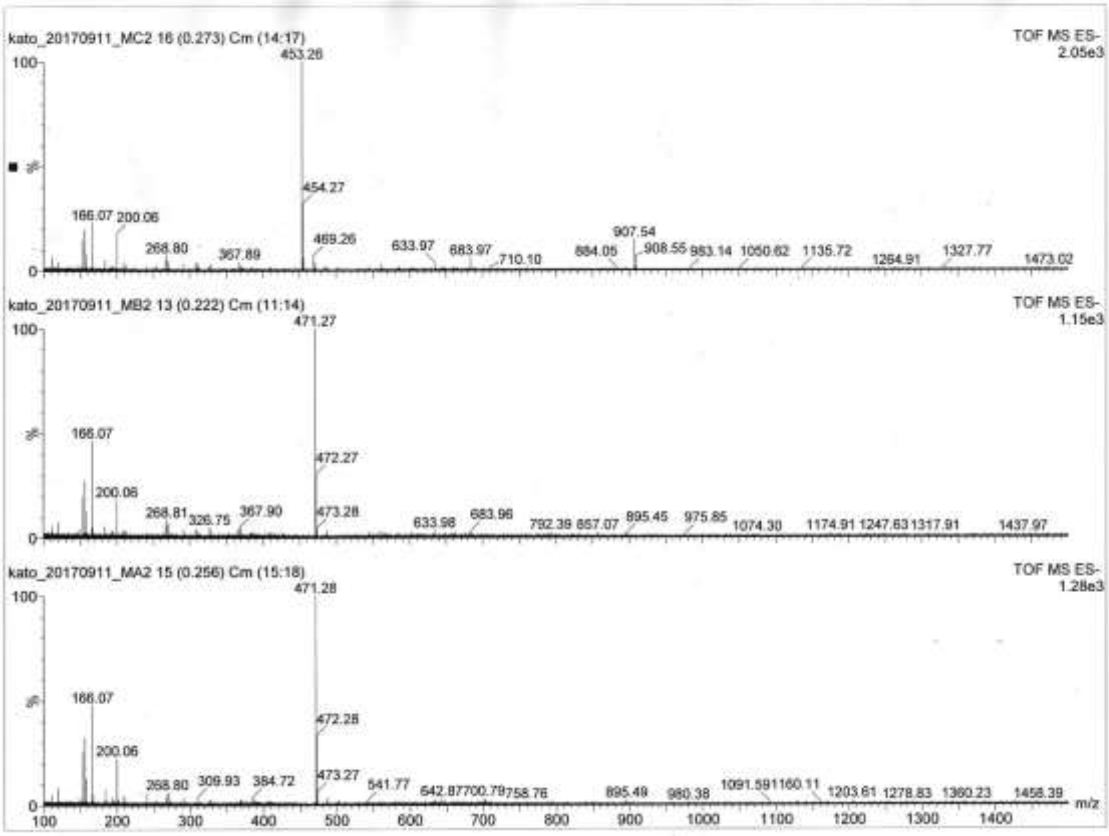

S Figure 7. ESI-MS (negative) analysis of macrocarpal A-C. Top: macrocarpal C, middle: macrocarpal B, bottom: macrocarpal A.
